# *Salmonella* Pathogenicity Island 2 Is Expressed Prior to Penetrating the Intestine

**DOI:** 10.1371/journal.ppat.0010032

**Published:** 2005-11-18

**Authors:** Nat F Brown, Bruce A Vallance, Brian K Coombes, Yanet Valdez, Bryan A Coburn, B. Brett Finlay

**Affiliations:** 1 Michael Smith Laboratories, University of British Columbia, Vancouver, British Columbia, Canada; 2 Division of Gastroenterology, BC Children's Hospital, Vancouver, British Columbia, Canada; Tufts University School of Medicine, United States of America

## Abstract

*Salmonella enterica* serovar Typhimurium is a facultative intracellular pathogen that causes disease in mice that resembles human typhoid. Typhoid pathogenesis consists of distinct phases in the intestine and a subsequent systemic phase in which bacteria replicate in macrophages of the liver and spleen. The type III secretion system encoded by *Salmonella* pathogenicity island 2 (SPI-2) is a major virulence factor contributing to the systemic phase of typhoid pathogenesis. Understanding how pathogens regulate virulence mechanisms in response to the environment, including different host tissues, is key to our understanding of pathogenesis. A recombinase-based in vivo expression technology system was developed to assess SPI-2 expression during murine typhoid. SPI-2 expression was detectable at very early times in bacteria that were resident in the lumen of the ileum and was independent of active bacterial invasion of the epithelium. We also provide direct evidence for the regulation of SPI-2 by the *Salmonella* transcription factors *ompR* and *ssrB* in vivo. Together these results demonstrate that SPI-2 expression precedes penetration of the intestinal epithelium. This induction of expression precedes any documented SPI-2-dependent phases of typhoid and may be involved in preparing *Salmonella* to successfully resist the antimicrobial environment encountered within macrophages.

## Synopsis

Typhoid fever is a disease caused by specific *Salmonella* strains and is a significant cause of mortality in many regions of the developing world. Following a person's ingestion of *Salmonella,* the bacteria initially colonize the intestine, which they subsequently breach to reside in immune cells of the liver and spleen. The ability to survive inside immune cells directly contributes to the ability of *Salmonella* to cause typhoid, and is conferred upon *Salmonella* by the so-called *Salmonella* pathogenicity island 2 (SPI-2) type III secretion system. Previous work has shown that while SPI-2 is specifically turned on inside host cells, it is not active when grown in typical laboratory medium. Owing to these facts, it has been hypothesized that *Salmonella* specifically turn on SPI-2 inside host cells after breaching the host intestine. The researchers developed a sensitive system in *Salmonella* to test this hypothesis using a mouse model of typhoid. Interestingly, SPI-2 was specifically turned on before *Salmonella* breached the intestine, suggesting that SPI-2, which is integral to virulence, is active in a preemptive fashion to allow *Salmonella* to survive within the immune system.

## Introduction

*Salmonella* is a Gram-negative bacterial pathogen that causes substantial morbidity and mortality worldwide. Human-adapted serovars cause typhoid, a systemic and life-threatening infection, while non-human-adapted serovars commonly cause enteritis. Following ingestion of contaminated food or water, the pathogenesis of both typhoid and *Salmonella* enteritis begins with an intestinal phase, while only typhoid progresses to a systemic phase. The intestinal phase of typhoid involves colonization of the intestine and penetration of the intestinal epithelium through two separate mechanisms. The first involves active bacterial invasion [1], and the second involves passive uptake of *Salmonella* during dendritic cell (DC) sampling of luminal microflora [2,3]. Once *Salmonella* has penetrated the intestinal epithelium, the systemic phase of typhoid begins by dissemination from the intestine via the lymphatics followed by colonization of macrophages of the liver and spleen [4,5]. The niche occupied by *Salmonella* within these cells is a membrane-bound compartment termed the *Salmonella*-containing vacuole. Much of our understanding of typhoid pathogenesis has come from mice infected with *S. enterica* serovar Typhimurium, which models human typhoid in several respects.

The current understanding of typhoid pathogenesis suggests that distinct virulence systems operate during the intestinal and systemic phases of infection and that these virulence systems display little overlap in their spatiotemporal activation. These virulence factors are type III secretion systems (T3SSs) that translocate numerous *Salmonella* virulence proteins (termed effectors) directly into the host cell, where they alter various host cell processes [6]. The T3SS encoded by *Salmonella* pathogenicity island 1 (SPI-1) allows *Salmonella* to invade non-phagocytic cells and penetrate the intestinal epithelium, and is the major factor involved during the intestinal phase of typhoid [7–9]. SPI-1 mutant *Salmonella* Typhimurium is fully virulent when inoculated intraperitoneally into mice, indicating that the role played by SPI-1 is limited to the intestinal phase of *Salmonella* infection [9]. In contrast, *Salmonella* pathogenicity island 2 (SPI-2) mediates *Salmonella* replication within macrophages at systemic sites, and SPI-2 mutant *Salmonella* is avirulent when inoculated intraperitoneally [10–12]. Studies of *Salmonella*-induced enteritis have found that the role of SPI-2 in the intestine is subtle when compared to the role of SPI-1 [13–16]. However, it should be noted that *Salmonella*-induced enteritis and typhoid are distinct diseases involving different intestinal pathologies, and as such the pathogenesis of the diseases is not directly comparable. In particular, typhoid does not typically involve significant intestinal inflammation [16]. This has led to models in which *Salmonella* employs a two-tiered expression of virulence systems that correspond to the biphasic pathogenesis of typhoid, with SPI-1 mediating intestinal pathogenesis and SPI-2 mediating systemic pathogenesis.

While it is clear that SPI-2 is a major virulence factor leading to mortality in murine typhoid, our current understanding of its molecular function is limited. Its primary role appears to be subversion of host cell membrane traffic, allowing replication of intracellular *Salmonella* [17]. Consistent with its role during intracellular growth, in vitro studies have shown that expression of SPI-2 is induced inside host cells and in culture medium that mimics the environment of the *Salmonella*-containing vacuole [18–20]. In vitro, SPI-2 expression is regulated by the SPI-2-encoded regulatory system SsrA/B [18,19], and efficient transcription of *ssrAB* requires the regulator OmpR [21]. Recent work by Hensel and colleagues has shown that the SPI-2 T3SS is typically expressed as one apparatus per bacterium, during growth in host cells or during inducing in vitro culture [22].

Countless studies have shown that bacteria regulate their expression profile by sensing the extracellular environment and responding accordingly. As an environment in which to study bacterial gene expression, the mammalian host poses unique challenges because of its complexity and dynamic nature. Consequently, analysis of the spatiotemporal expression pattern of virulence genes in vivo has rarely been undertaken. Of particular interest to the field of *Salmonella* research is the expression pattern of SPI-2 during typhoid because it is the central virulence mechanism and is considered to play a role specific to a particular phase of pathogenesis. Based on this, we hypothesized that SPI-2 expression would be confined to the peripheral lymphoid tissues, spleen, and liver. To address this, we examined the spatiotemporal expression pattern of three SPI-2 promoters during experimental murine typhoid. In contrast to the current model of SPI-2-mediated pathogenesis that argues for exclusive intracellular expression, we found that expression of SPI-2 occurs during initial stages of pathogenesis in the lumen of the intestine.

## Results

### SPI-2 Gene Expression In Vitro Assessed Using RIVET

Recombinase-based in vivo expression technology (RIVET) is an exquisitely sensitive reporter of gene expression. This system involves the construction of a transcriptional fusion to a site-specific recombinase, which mediates the loss of a selectable genetic marker (a process called resolution) [23]. This approach has been applied to elucidate the spatiotemporal expression patterns of the toxin-coregulated pilus and cholera toxin during *Vibrio cholerae* infection of mice [24]. We designed and constructed a *Salmonella* Typhimurium strain possessing all the genetic requirements for RIVET and *lacZ* fusion analysis of P*_sseA_* (see Materials and Methods). The ability of this strain to function as a RIVET reporter of P*_sseA_* activity was initially assessed during in vitro growth in media that have previously been shown to induce (LPM medium) or not induce (Luria-Bertani [LB] medium) expression from SPI-2 promoters (Figure 1). P*_sseA_* activity was assessed using RIVET and β-galactosidase activity as well as by monitoring cytoplasmic levels of SseB, a protein expressed from P*_sseA_*. A low level of P*_sseA_* activity was detected for each output in the non-inducing LB medium whereas high levels were detected from bacteria cultured in the inducing LPM medium, indicating that RIVET correlates well with standard methods. Additionally, RIVET was considerably more sensitive at detecting P*_sseA_* activity than *lacZ* transcriptional fusion and immunoblotting for native SseB levels. This was most obvious when the number of bacteria in the culture was below the sensitivity of conventional reporter systems. Furthermore, the high degree of sensitivity of RIVET was deemed important for the study of the monocopy expression level of the SPI-2 T3SS. Using RIVET, we also confirmed the role of SsrB and OmpR in regulating SPI-2 gene expression (Figure 1D).

**Figure 1:**
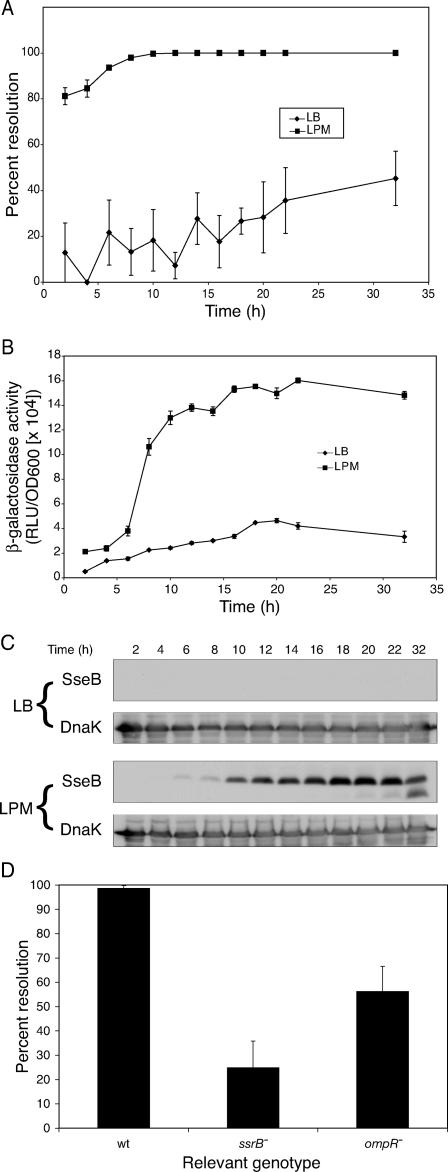
Expression from P*_sseA_* during Growth of *Salmonella* in Inducing and Non-Inducing Culture Media (A–C) The wild-type *Salmonella* RIVET strain (NB25) was inoculated into LPM (inducing) and LB (non-inducing) media at 1/100 of the culture volume, and cultures were incubated with shaking at 37 °C. At the indicated time points, samples were taken for measurement of culture OD_600_ (unpublished data), colony-forming units per millilitre (unpublished data), β-galactosidase activity, percent resolution, and native SseB levels. The results shown for percent resolution (A) and β-galactosidase activity (B) are the mean ± standard error of the mean from three independent experiments. The levels of SseB associated with bacteria were detected by immunoblotting (C). Protein loading was normalized to culture OD_600_ and was confirmed by immunoblotting for the abundantly expressed cytoplasmic protein DnaK. (D) Wild-type (NB25), *ssrB* ^−^ (NB7), and *ompR* ^−^ (NB15) were grown in LPM medium and percent resolution was determined from the cultures at 10 h.

*Salmonella* infection of cultured mammalian cells is a model that is frequently used to approximate conditions encountered during infection in vivo. We determined the expression from P*_sseA_* at 1 h following uptake into various mammalian cells using the RIVET system (Figure 2). In HeLa cells (epithelial), where bacteria actively induce their own uptake using the SPI-1 T3SS, substantial induction of expression from P*_sseA_* occurred after 1 h in an SsrB- and an OmpR-dependent fashion. This demonstrates that SPI-2 is activated rapidly following SPI-1-mediated *Salmonella* invasion of an epithelial cell. The role of SPI-2 in mediating replication within macrophages has been thoroughly documented [17], and DCs are also a potentially important cell type encountered by *Salmonella* in vivo [2,3]. We therefore determined the kinetics of P*_sseA_* induction in RAW264.7 murine macrophages and murine DCs. A significant induction of SsrB- and OmpR-dependent P*_sseA_* expression could be detected within 1 h of *Salmonella* being added to either cell type (Figure 2).

**Figure 2:**
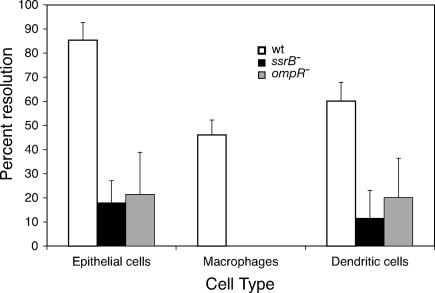
Expression from P*_sseA_* during Infection of Mammalian Cells In Vitro The human epithelial cell line HeLa, murine macrophage cell line RAW264.7, and bone-marrow-derived DCs were infected with wild-type (NB25), *ssrB ^−^* (NB7), and *ompR ^−^* (NB15) RIVET strains as described in Materials and Methods. At 1 h post-infection intracellular bacteria were recovered and the percent resolution was determined.

### SPI-2 Gene Expression In Vivo Assessed Using RIVET

Once it was established that the RIVET system was sensitive and specific, the RIVET system was used to determine the spatiotemporal expression pattern of P*_sseA_* during infection of mice. To facilitate the synchronized arrival of a bacterial load in the distal ileum, the inoculum was delivered into a single loop constructed in the ileum of each mouse tested (see Materials and Methods). At times ranging from 15 min to 4 h following inoculation, the liver, spleen, mesenteric lymph nodes, and ileal loop were dissected, homogenized, and plated to determine the degree of P*_sseA_* induction using RIVET (Figure 3A). These results clearly demonstrated that expression from P*_sseA_* was induced within 15 min following inoculation and that this induction occurred within the ileum. RIVET reports gene expression in an irreversible fashion, and data on expression at systemic sites and later time points was not considered reliable as the majority of bacteria had undergone resolution within 15 min. We also assessed *ssrB^−^* and *ompR^−^* strains for their ability to induce expression from P*_sseA_* within the ileum 15 min following inoculation (Figure 3B). These data showed that both regulators play a significant role in the observed induction in the ileum and directly showed the involvement of these regulators in the expression of SPI-2 in vivo.

**Figure 3:**
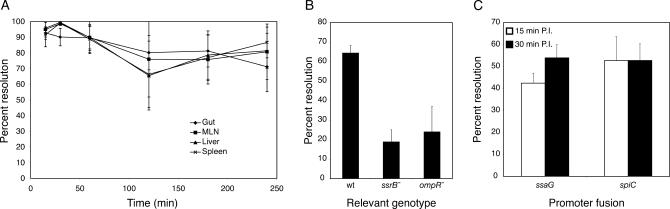
Kinetics of SPI-2 Expression during Murine Typhoid (A) Mice were infected with the wild-type P*_sseA_* RIVET strain (NB25) as described in Materials and Methods. At the indicated times following inoculation, the ileal loops, mesenteric lymph nodes, spleen, and liver were taken and assessed for expression from P*_sseA_* by determining the percent resolution. The results shown are the mean ± standard error of the mean from three independent experiments. (B) Mice were infected with the wild-type (NB25), *ssrB* ^−^ (NB7), and *ompR* ^−^ (NB15) P*_sseA_* RIVET strains, and at 15 min following inoculation, the ileal loop was removed and homogenized, and the percent resolution of the infecting *Salmonella* was determined. (C) Mice were infected with the wild-type P*_spiC_* (NB33) and P*_ssaG_* (NB31) RIVET strains for 15 and 30 min, the ileum was removed and homogenized, and the percentage resolution determined. The results shown are the mean ± standard error of the mean for three independent experiments.

The experiments described above showed that P*_sseA_,* the promoter driving expression of the SPI-2 T3SS translocon and effectors, is active within 15 min of inoculation into ileal loops. To see if the promoters driving expression of the remaining components of the SPI-2 T3SS were active on a similar timescale, we constructed strains for RIVET analysis of transcription from P*_spiC_* and P*_ssaG_*. These strains were confirmed to have the typical SPI-2 expression characteristics during in vitro culture, including being dependent on the SPI-2-encoded transcription factor SsrB (data not shown). When these strains were inoculated into mouse ileal loops, expression after 15 min from both P*_spiC_* and P*_ssaG_* could be observed (Figure 3C), similar to that of previous experiments with P*_sseA_*. These results showed that all SPI-2 T3SS components are expressed in a large proportion of *Salmonella* within 15 min of arriving in the ileum.

The short time frame in which SPI-2 expression was induced in vivo prompted us to investigate the location of the bacteria that had induced SPI-2 expression. Specifically, we wanted to know whether *Salmonella* was located within host cells when SPI-2 expression was induced. As SPI-1 mediates the major route of epithelial penetration [2,8], we tested expression from P*_sseA_* in a SPI-1 mutant (*invA* ^−^) in vivo. At 15 min following inoculation, the induction of expression from P*_sseA_* was independent of SPI-1 (Figure 4A), strongly suggesting that the SPI-2 expression we were observing was occurring in the lumen of the intestine. To further test this, we directly determined the localization of wild-type *Salmonella* 15 min following inoculation into ileal loops using confocal microscopy. As expected, the vast majority of bacteria were not located within host cells but rather were associated with the apical surface of the epithelium (Figure 4B). These data are inconsistent with the observed induction of SPI-2 occurring exclusively in an intracellular compartment in vivo.

**Figure 4:**
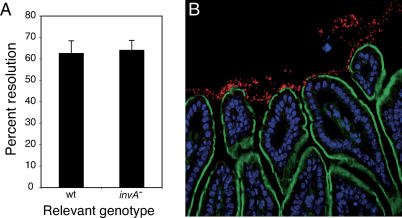
*Salmonella* Induces Expression of SPI-2 in the Lumen of the Ileum (A) Mouse ileal loops were infected with either wild-type or *invA* ^−^ P*_sseA_* RIVET strains for 15 min before the loops were removed and homogenized, and the percent resolution was determined. (B) Mouse ileal loops were infected with wild-type *Salmonella* for 15 min before being removed, fixed, stained, and analyzed by confocal microscopy with an oil immersion 40× 1.3 N.A. objective. A representative image is displayed showing host cell nuclei in blue, actin in green, and *Salmonella* in red.

To test whether *Salmonella* association with the cytoplasmic membrane of a host cell initiates expression from P*_sseA_,* we infected HeLa cells in vitro with noninvasive *invA* ^−^ *Salmonella*. Extracellular, cell-associated *invA* ^−^ *Salmonella* at 1 h post-infection had not induced significant expression from P*_sseA_* (3.54% ± 3.01% resolution). In contrast, internalized wild-type *Salmonella* controls showed a high level of expression (75.10% ± 5.75% resolution). This is consistent with cell culture medium providing a non-inducing environment for SPI-2 expression (data not shown) and shows that the host cell plasmalemma does not induce SPI-2 expression. This suggested that a stimulus for expression from P*_sseA_* exists in the luminal contents of the small intestine. We therefore conducted experiments to address this hypothesis. Wild-type, *ssrB* ^−^, and *ompR* ^−^ RIVET strains were incubated for up to 30 min at 37 °C with contents collected from mouse small intestine and then resolution was measured. No resolution was observed for any strain, suggesting that the stimulus inducing expression of SPI-2 in the intestine is not capable of being extracted with naive luminal contents of the small intestine.

## Discussion

By using a sensitive methodology and testing earlier time points than have been presented in previous studies of SPI-2 function, we have established that SPI-2 genes are expressed very early in the intestine. The data presented above are inconsistent with the currently held view that the initial induction of SPI-2 expression occurs in response to an intracellular environment. Our attempts to confirm the RIVET expression data using β-galactosidase reporters to measure SPI-2 expression in vivo failed to detect sufficient signal to reliably report SPI-2 expression (data not shown). However, extensive controls were performed in vitro, where sufficient numbers of bacteria can be analyzed to detect β-galactosidase reporters as well as SPI-2 proteins by western blotting. These control experiments confirmed that RIVET was a sensitive and specific reporter of SPI-2 expression. The failure of β-galactosidase to accurately report on SPI-2 expression in vivo is most likely a reflection of low bacterial numbers in the examined tissues. This highlights a major advantage of RIVET in its capacity to report on gene expression from small populations of bacteria.

Previous work has focused on mimicking the intracellular environment encountered by *Salmonella* to define the cues for inducing expression of SPI-2. Our results show that SPI-2 is induced in the gut lumen, prior to encountering an intracellular environment. We have investigated signals present in the small intestine as potential cues for SPI-2 expression and have determined that intestinal SPI-2 induction is not a result of association with host cell plasmalemma or luminal contents from the small intestine of uninfected mice. Additionally, we have been able to rule out low oxygen tension as a cue for SPI-2 induction in the intestine (data not shown). Our data are compatible with a rapid host response to *Salmonella* acting as a cue for the induction of SPI-2 expression. One such response would be the secretion of antimicrobial products by paneth cells, specialized epithelial cells that reside at the base of the crypts and respond to antigenic stimuli including *Salmonella* [25]. However, experiments with *mmp7* ^−/−^ mice, which are deficient in paneth cell products, have indicated that these products are not a stimulus for SPI-2 expression in vivo (data not shown). Other potential host responses acting as a stimulus for SPI-2 expression will be a topic of future investigation.

Previous studies on SPI-2 and its translocated effectors have almost exclusively focused on the role played during the systemic phase of typhoid, and consequently little is known about the potential actions of SPI-2 during the intestinal phase of typhoid. Others have shown that SPI-2 mutant *Salmonella* colonizes the caecum and Peyer's patches to a lesser extent than wild-type *Salmonella* during typhoid [19]. This, together with our data on expression, firmly establishes a role for SPI-2 prior to colonization of systemic sites during typhoid. It is important to consider the localization of intestinal *Salmonella* in order to speculate on the role SPI-2 could be playing at this location. Although the vast majority of bacteria are present in the luminal space of the intestine, it is well established that *Salmonella* transits through epithelial cells and can reside within sub-epithelial phagocytic cells. Given that type III secretion mediates the direct delivery of effector proteins into the host cell cytosol [6] and that the system encoded by SPI-2 allows *Salmonella* to replicate within macrophages [17], this suggests that the location where SPI-2 is actually functioning is within phagocytic cells of the intestine. It is likely that SPI-2 expression initiated in the intestinal lumen primes *Salmonella* for residency in intestinal phagocytic cells prior to the bacteria reaching this niche. We do not conclude that SPI-2 enables replication of luminal *Salmonella*.

The rapid induction of SPI-2 in the lumen of the ileum suggests that for *Salmonella* to initiate a successful infection of sub-epithelial and systemic sites, it must be prepared for the harsh environment of the macrophage endosomal system prior to the encounter. This finding may establish a paradigm for all pathogens where preemptive synthesis of important virulence factors occurs prior to the transition from colonization of a mucosal surface to colonization of systemic sites. We also speculate that functions mediated by SPI-2 during early stages of infection may not be limited to the establishment of a *Salmonella*-containing vacuole that supports bacterial replication. Such possibilities include enhancing dissemination to systemic sites. By identifying additional SPI-2-dependent functions at these early intestinal stages of typhoid pathogenesis, we hope to elucidate key mechanisms that facilitate the successful parasitic lifestyle of *Salmonella*.

## Materials and Methods

### Bacterial strains and plasmids.

Bacterial strains and plasmids are shown in Table 1. All *Salmonella* strains used in this study were derived from the wild-type strain SL1344. All bacteria were routinely cultured in LB medium. LPM medium (pH 5.8) has been described previously [26]. Kanamycin was used at 50 μg ml^−1^, chloramphenicol at 10 μg ml^−1^, and ampicillin at 100 μg ml^−1^. *Salmonella* RIVET strains were always grown in LB medium containing ampicillin and chloramphenicol prior to conducting experiments.

**Table 1:**
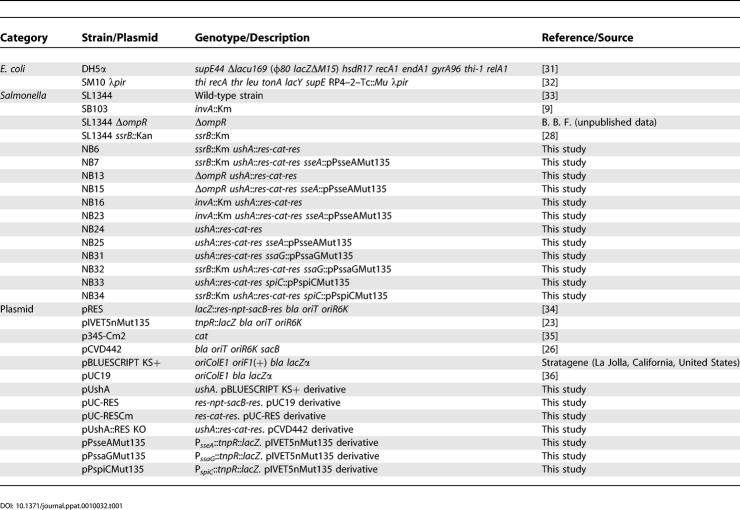
Bacterial Strains and Plasmids Used in This Study

For RIVET studies, it is desirable to insert the resolvable cassette *(res-*marker*-res)* into a neutral site within the genome. *ushA* is a silent gene in *Salmonella* Typhimurium because of an inactivating S139Y substitution in the expressed protein product [27], and as such this was considered to be a good site to insert the resolvable cassette for RIVET. A region of approximately 1.8 kb spanning *ushA* was amplified by PCR using the oligonucleotides ushako-f (5′ GCAACTAGTGCGATGTTGGAGATAGTAGGATGTG 3′) and ushako-r (5′ GATGTCGACCCTCACCATTTGGCGTAAACG 3′) and was cloned into pBLUESCRIPT KS+ using SpeI and SalI to create pUshA. A resolvable cassette mediating resistance to chloramphenicol was constructed by subcloning the *res-npt-sacB-res*-containing SphI fragment into pUC19 to create pUC-RES. The *npt-sacB* portion was replaced with *cat* to give pUC-RESCm. The *res-cat-res* cassette was then inserted into the middle of *ushA* in pUshA, and then the *ushA*::*res-cat-res* allele was cloned into pCVD442 using SacI and KpnI to give pUshA::RES KO. Using this plasmid, the *ushA*::*res-cat-res* allele was introduced into SL1344 and SL1344 Δ*ompR* using standard allelic exchange methodologies [28] to give strains NB24 and NB13, respectively. NB24 was shown to have no detectable defect in virulence when inoculated intraperitoneally into mice by either a time-to-death assay (N. F. B. and B. A. V., unpublished data) or competitive index (B. K. C. and M. Wickham, unpublished data). *Salmonella* strains NB6 and NB16 were created by P22 generalized transduction of *ssrB*::Km and *invA*::Km alleles into NB24, respectively.

Transcriptional fusions were generated as follows. An approximately 1.5-kb region upstream of *sseA* was amplified by PCR using oligonucleotides pssea-f (5′ ATACTCGAGCGTATTCTTCATTTTCATCGGTG 3′) and pssea-r (5′ ATACAATTGCCCTTTCAGCAAGCTGTTGAC 3′) and cloned into pIVET5nMut135 using XhoI and MfeI. Other reporter strains were generated using the same strategy using oligonucleotides pssagfu-f (5′ ATGCTCGAGAATTACCTCCGTTAGCCTGAC 3′) and pssagfu-r (5′ ATGCAATTGCTGCCTGGTGCGCCATGTG 3′) for P*_ssaG_* and oligonucleotides pssab-f (5′ ATTCTCGAGTCGGCGTGCAATTTGAAGG 3′) and pssab-r (5′ ATACAATTGCCAGCATGAATCCCTCCTCAGAC 3′) for P*_spiC_*. The resulting reporter plasmids were conjugated into the desired *Salmonella* strains from *Escherichia coli* SM10 λ*pir*. The resulting strains had the reporter plasmid integrated into the chromosome by homologous recombination, resulting in a merodiploid genotype for the promoter region being studied.

### Gene expression reporter assays.

Resolution assays were performed by plating serial dilutions of sample material onto LB ampicillin plates and incubating these overnight at 37 °C. The following day, plates with between 50 and 200 colonies were replica-plated onto LB ampicillin plates and LB ampicillin chloramphenicol plates, which were incubated overnight at 37 °C. Colonies that grew on the ampicillin only plates, but not on the ampicillin chloramphenicol plates were considered to have undergone the resolution event. β-galactosidase assays were performed as previously described [29] using Galacto-Star chemiluminescent substrate (Applied Biosystems, Foster City, California, United States) and were read using a Spectrafluor Plus (Tecan, Mannedorf, Switzerland) in luminescence mode.

### Antibodies and reagents.

Antibodies to SseB have been described previously [30] and were used at a concentration of 1:1,000, and a monoclonal antibody to DnaK (Stressgen Biotechnologies, Victoria, British Columbia, Canada) was used at a concentration of 1:2,000. Anti-*Salmonella* Typhimurium LPS antiserum (Difco, Becton-Dickinson, Franklin Lakes, New Jersey, United States) was used at a concentration of 1:200. HRP-labeled anti-mouse and anti-rabbit antibodies were used at a concentration of 1:5,000 and were purchased from Jackson Immunoresearch Laboratories (West Grove, Pennsylvania, United States). Alexafluor 568–labeled anti-rabbit antibodies (Molecular Probes, Eugene, Oregon, United States) were used at a concentration of 1:200. Alexafluor 488–labeled phalloidin and DAPI were purchased from Molecular Probes.

### Cell culture and infection of cultured cells.

HeLa and RAW264.7 cells were cultured using DMEM containing 10% FCS. Bone-marrow-derived DCs were derived as previously described [31]. Briefly, 2.5 × 10^6^ bone marrow cells were cultured in 10 ml of Iscove's Modified Dulbeco's Medium supplemented with 10% FCS, GM-CSF (20 ng/ml), and IL-4 (10 ng/ml). The cells were harvested after 6 d of culture, and DCs were purified using anti-CD11c conjugated MACS microbeads and magnetic separation columns (Miltenyi Biotec, Bergisch Gladbach, Germany). The purity of DCs was assessed by FACS analysis and ranged from 70% to 90% CD11c^+^. Cells were incubated in an atmosphere containing 5% CO_2_ at 37 °C.

HeLa cells were infected with invasive *Salmonella* prepared according to previous studies [32]. RAW264.7 and DCs were infected with stationary phase *Salmonella* opsonized in 20% normal human serum for 30 min. An incubation of 10 min at 37 °C in 5% CO_2_ was performed once bacteria were applied to cell cultures to allow for internalization. Following this, the infecting medium was aspirated, cells were washed four times with PBS, fresh cell culture medium containing gentamicin (100 μg ml^−1^) was added, and incubation at 37 °C in 5% CO_2_ was continued. At 1 h post-infection, cell culture medium was removed and the cells were again washed four times with PBS. The cells were then lysed in a solution of Triton X-100 (1% v/v) and sodium dodecyl sulfate (0.1% w/v) to release intracellular bacteria. Samples were diluted and assayed for resolution.

### Mouse infections.

Female C57BL/6 mice (6–10 wk of age) were purchased from Jackson Laboratory (Bar Harbor, Maine, United States), and housed in the animal facility at the University of British Columbia in direct accordance with guidelines drafted by the University of British Columbia's Animal Care Committee and the Canadian Council on the Use of Laboratory Animals. For ileal loop experiments, bacterial inocula of approximately 10^7^ colony-forming units were prepared in 100 μl, and the resolution status of the strain was confirmed directly before inoculation. Ileal loop experiments were modified from those previously described [1]. In brief, mice were anaesthetized by intraperitoneal injection of ketamine and xylazine. Following a midline abdominal incision, the small bowel was exposed and the bowel was ligated twice, close to the cecum, to create a loop approximately 4 cm in length into which the inoculum was injected. The bowel was then returned to the abdominal cavity and the incision closed with discontinuous sutures. At given time points, the mice were euthanized and tissues collected for bacterial enumeration and RIVET. The intestinal lumen was rinsed with PBS to remove non-adherent bacteria. Tissues were homogenized in PBS using a Polytron homogenizer (Kinematica, Lucerne, Switzerland).

### Immunohistochemistry.

Tissues were fixed for 3 h in 3% paraformaldehyde, and washed three times with PBS prior to cryoembedding and sectioning. Sections were fixed in acetone at −20 °C for 10 min and then air dried at room temperature. Tissue sections were outlined with a wax pen and blocked 30 min in 10% goat serum in PBS-BSA at room temperature. Sections were then washed three times in PBS-BSA prior to incubation for 30 min at room temperature with anti-*Salmonella* LPS antiserum. Sections were then washed three times in PBS-BSA prior to incubation with appropriate Alexafluor 568–conjugated anti-rabbit antibodies. Sections were then washed three times in PBS-BSA, incubated with Alexafluor 488–labeled phalloidin, washed a further three times in PBS-BSA, and then incubated in DAPI. Imaging was performed on a Nikon (Tokyo, Japan) TE2000 inverted microscope equipped with a Bio-Rad (Hercules, California, United States) Radiance 2000 confocal scan head and a two-photon laser source using a plan apochromat 40× 1.3 N.A. objective.

## Abbreviations

DC: dendritic cell
LB: Luria-Bertani
RIVET: recombinase-based in vivo expression technology
SPI-1: *Salmonella* pathogenicity island 1
SPI-2: *Salmonella* pathogenicity island 2
T3SS: type III secretion system
